# WWC2 is an independent prognostic factor and prevents invasion *via* Hippo signalling in hepatocellular carcinoma

**DOI:** 10.1111/jcmm.13281

**Published:** 2017-08-16

**Authors:** Yijun Zhang, Shumei Yan, Jiewei Chen, Caixia Gan, Dong Chen, Yan Li, Jiahuai Wen, Joachim Kremerskothen, Shilu Chen, Jiangbo Zhang, Yun Cao

**Affiliations:** ^1^ Collaborative Innovation Center for Cancer Medicine State Key Laboratory of Oncology in South China Sun Yat‐sen University Cancer Center Guangzhou China; ^2^ Department of Pathology Sun Yat‐Sen University Cancer Center Guangzhou China; ^3^ Department of Urology Sun Yat‐sen University Cancer Center Guangzhou China; ^4^ Department of Oncology Tumor Angiogenesis and Microenvironment Laboratory (TAML) First Affiliated Hospital of Jinzhou Medical University Jinzhou China; ^5^ Department of Breast Oncology The Second Affiliated Hospital of Guangzhou University of Chinese Medicine Guangzhou China; ^6^ Internal Medicine D Department of Nephrology, Hypertension and Rheumatology University Hospital Muenster Muenster Germany

**Keywords:** WWC2, YAP, LATS2, hepatocellular carcinoma, hippo pathway

## Abstract

WWC family proteins negatively regulate HEK293 cell proliferation and organ growth by suppressing the transcriptional activity of Yes‐associated protein (YAP), a major effector of the Hippo pathway. The function of the scaffolding protein WWC1 (also called KIBRA) has been intensively studied in cells and animal models. However, the expression and clinicopathologic significance of WWC2 in cancer are poorly characterized. This study aimed to clarify the biological function and mechanism of action of WWC2 in hepatocellular carcinoma (HCC). Retrospective analysis revealed WWC2 was significantly down‐regulated in 95 clinical HCC tissues compared to the paired adjacent non‐cancerous tissues. Moreover, loss of WWC2 expression was significantly associated with advanced clinicopathological features, including venous infiltration, larger tumour size and advanced TNM stage. Positive WWC2 expression was associated with significantly better 5‐year overall survival, and WWC2 was an independent prognostic factor for overall survival in HCC. Moreover, we confirmed WWC2 inhibits HCC cell invasive ability *in vitro*. Elevated YAP expression was also observed in the same cohort of HCC tissues. Pearson's correlation coefficient analysis indicated WWC2 expression correlated inversely with nuclear YAP protein expression in HCC. Mechanistically, we confirmed overexpression of WWC2 suppresses the invasive and metastatic potential of HCC cells by activating large tumour suppressor 1 and 2 kinases (LATS1/2), which in turn phosphorylates the transcriptional co‐activator YAP. Overall, this study indicates WWC2 functions as a tumour suppressor by negatively regulating the Hippo signalling pathway and may serve as a prognostic marker in HCC.

## Introduction

Hepatocellular carcinoma (HCC) is the fifth most common cancer and third leading cause of cancer‐related deaths worldwide 1, and its incidence is increasing. Surgical reaction and liver transplantation are currently the best curative options for HCC. However, recurrence or metastasis is relatively common after resection 2. Multiple signalling pathways contribute to the development of HCC. Recently, the Hippo pathway has been found to be involved in cell size regulation, cell proliferation, cell death and tumorigenesis of HCC 3, 4, 5, 6.

The Hippo pathway contains a number of tumour suppressors and oncogenes. Core components and upstream regulators such as LATS1/2 and mammalian Ste20‐like kinases 1 and 2 (MST1/2) predominantly function as tumour suppressors, whereas PDZ‐binding Motif (TAZ), Yes‐associated protein (YAP) and (TEA)‐domain family members (TEADs) promote oncogenic events 7, 8, 9. YAP is a pivotal effector of the Hippo pathway 10, 11. Previous studies identified YAP was an independent prognostic marker, and inactivation of the Hippo pathway was significantly associated with poorer prognosis in HCC 12. Moreover, overexpression of YAP induces the epithelial–mesenchymal transition (EMT), growth factor‐independent cell proliferation and oncogenesis 13, 14, 15. In mammals, several tumour suppressors (Mst1/2, Sav1/WW45, Lats1/2, Mob1) regulate the transcriptional co‐activator YAP, forming a kinase cascade that culminates in phosphorylation and inactivation of YAP 16, 17, 18. Unphosphorylated YAP localizes to the nucleus, where it serves as a co‐activator for TEAD DNA‐binding transcription factors 19, 20. Although the Hippo pathway is well characterized, the ability of upstream regulators of this pathway to function as oncogenic drivers in HCC remains largely unknown.

Recent studies in *Drosophila melanogaster* and mammalian cells identified WW‐and‐C2‐domain‐containing protein (WWC) family proteins as regulatory elements of the Hippo pathway 21, 22. WWC family members include WWC1 (also known as KIBRA), WWC2 and WWC3 23. Similarly to the other two WWC family members, WWC2 contains two amino terminal WW domains that mediate binding to target proteins harbouring L/PPxY motifs, as well as an internal C2 domain for membrane association 22. WWC proteins can negatively regulate Hippo signalling by activating the LATS1/2 kinases, which in turn phosphorylate YAP and prevent its nuclear import, which negatively regulates cell proliferation and regulates tissue growth in mammalian cells 21, 22, 24, 25. Wennmann and colleagues identified WWC proteins enhance phosphorylation of LATS1/2 and YAP, reduce the transcriptional activity of YAP and impair cell proliferation in HEK293 cells 22. Although the function of WWC1 has been studied intensively in cells and animal models, our understanding of the expression, biological behaviour and molecular mechanisms of action of WWC2 remains limited, particularly in human cancer.

In view of the ability of WWC2 to regulate the transcriptional activity of YAP by activating LATS1/2 in HEK293 cells, we suggested that WWC2 may also inhibit cell invasion in HCC by negatively regulating Hippo signalling. In this study, we aimed to explore the relationship between WWC2 and the clinicopathologic features of HCC and define the role of WWC2 in regulation of the Hippo signalling pathway in HCC.

## Materials and methods

### Ethics statement

The study was approved by the Institute Research Medical Ethics Committee of Sun Yat‐sen University, and informed consent (written or verbal) was obtained from the patients in this study for retrospective analysis of tissue samples. All samples were anonymized.

### Clinical samples

Tumour samples and paired normal tumour‐adjacent samples (>2 cm distance from the margin of the resection) from 95 patients with HCC treated between 2000 and 2006 were obtained from the archives of the Department of Pathology, Sun Yat‐sen University Cancer Center, Guangzhou, China. The cases were selected based on the following criteria: pathological diagnosis of HCC; primary and curative tumour resection without pre‐operative or post‐operative anticancer treatment; and availability of resection tissue and follow‐up data. The HCC cohort included 83 (87.4%) males and 12 (12.6%) females with a mean age of 49.0 years. The patients were followed up every 3 months after surgery for the first year, every 6 months for the next 2 years, then annually; all patients were followed up for at least 2 years. The clinicopathologic features summarized in Table 1 include age, gender, hepatitis history, serum alpha‐fetoprotein (AFP) level, presence of cirrhosis, number of lesions, tumour size, level of tumour differentiation, tumour stage, extent of vascular invasion and capsule invasion. Tumour differentiation and stage were defined according to the tumour‐node‐metastasis (TNM) classification system of the American Joint Committee on Cancer/International Union Against Cancer. Overall survival was calculated from the date of diagnosis to the date of death. The other 24 fresh HCC tissues and adjacent non‐tumour tissues samples were obtained from 24 patients who underwent surgical resection for HCC at Sun Yat‐sen University Cancer Center. All samples were frozen in liquid nitrogen immediately after resection and stored at −80°C until use.

**Table 1:** Clinical characteristics and WWC2 expression for 95 cases of hepatocellular carcinoma

Characteristic	Patients	WWC2		*P‐*value
		Negative (%)	Positive (%)	
Age (years)				
<60	47	30	17	
≥60	48	31	17	0.939
Gender				
Male	83	54	29	
Female	12	7	5	0.650
HBV				
Absent	10	5	5	
Present	85	56	29	0.322
Cirrhosis				
Absent	15	7	8	
Present	80	54	26	0.122
Tumour size (cm)				
<5	24	11	13	
≥5	71	50	21	0.030^a^
Serum AFP (ng/ml)				
<400	57	37	20	
≥400	38	24	14	0.861
No. of tumour nodules				
1	81	50	31	
≥2	14	11	3	0.225
Differentiation				
Well	7	4	3	
Moderate	43	28	15	
Poor	45	29	16	0.919
Capsule invasion				
Absent	63	39	24	
Present	32	22	10	0.511
Venous infiltration				
Absent	71	40	31	
Present	24	21	3	0.006^a^
TNM stage				
I + II	67	35	32	
III + IV	28	26	2	0.001^a^

HCC, hepatocellular carcinoma; HBV, hepatitis B virus; AFP, alpha‐fetoprotein; TNM, tumour‐node‐metastasis.

a Statistically significant.

### Cell culture

Human HCC cell lines QGY‐7703, QGY‐7701, SMMC‐7721, MHCC‐97L, MHCC‐97H, HepG2, Bel‐7402 and Bel‐7404 and the human normal liver cell line L02 were purchased from the Cell Resource Center, Chinese Academy of Science Committee (Shanghai, China). Cells were cultured in RPMI‐1640 medium containing 10% foetal bovine serum at 37°C in a humidified atmosphere of 5% CO_2_.

### Plasmid construction and transfection

Expression plasmids for pCDNA‐3x‐Flag‐WWC2 (encoding a triple Flag‐tagged WWC2 protein and kanamycin resistance gene) and V180‐3x‐Flag (control vector, encodes only the 3x‐Flag tag and kanamycin resistance gene) were obtained from Kremerskothen 22. The sequences of the *WWC2* siRNA (Guangzhou Ruibo Co. Ltd, Guangzhou, China) were as follows: si‐h‐WWC2: GAGCCAGATTTGAGATGTA. The wild‐type (WT) LATS2 and various LATS2 mutant expression plasmids were obtained from Dr. Dong 21. Cells were transiently transfected with *WWC2* siRNA and various plasmids using Lipofectamine 2000 (Invitrogen, Carlsbad, CA, USA) according to the manufacturer's instructions.

### Immunohistochemistry

The paraffin‐embedded samples were serially cut into 4‐mm‐thick sections, de‐paraffinized in xylene, rehydrated through a graded alcohol series, immersed in 3% hydrogen peroxide for 10 min. to block endogenous peroxidase activity and subjected to antigen retrieval by pressure cooking for 3 min. in citrate buffer (pH 6.0). Then, the slides were incubated with 10% normal goat serum at room temperature for 30 min. to reduce non‐specific reactivity and then incubated with rabbit polyclonal anti‐WWC2 antibody (1:100; Abcam, Cambridge, MA, USA) or rabbit monoclonal anti‐YAP antibody (1:100, cat.14074; Cell Signaling Technology, Boston, MA, USA) overnight at 4°C. Slides were washed twice with PBS for 5 min., incubated with a secondary antibody (Envision; Dako, Glostrup, Denmark) for 1 hr at room temperature, washed twice with PBS for 5 min. and developed using 3,3‐diaminobenzidine (DAB). Finally, the sections were counterstained with Mayer's haematoxylin, dehydrated and mounted. Negative controls were prepared by replacing the primary antibody with normal murine IgG.

All sections were scored independently by two experienced pathologists. The labelling score was obtained by multiplying the score for the percentage of positively stained cells [‘0’ (<5%), ‘1’ (6–25%), ‘2’ (26–50%), ‘3’ (51–75%), ‘4’ (76–100%)] by the score for staining intensity [‘0’ (negative staining), ‘1’ (weak staining), ‘2’ (moderate staining) and ‘3’ (strong staining)] to give a final score for each section. Sections with a total score of ≥4 were defined as exhibiting positive staining.

Nuclear YAP positivity was scored by determining the percentage of positive nuclei regardless of cytoplasmic YAP expression and staining intensity.

### Western blotting

Western blotting was performed as previously described 26. Protein samples (50 μg) were separated by SDS‐PAGE (10%), transferred to a polyvinylidene fluoride (PVDF) membranes (Millipore, Billerica, MA, USA) and incubated overnight at 4°C with the following antibodies: YAP (1:500, cat. 14074), pYAP‐S127 (1:500, cat. 57706), vimentin (1:1,000, cat. 12826), snail (1:500, cat. 3895) (Cell Signaling Technology), Flag (1:5,000, cat. F3165), c‐myc (1:5,000, cat. M5546) (Sigma‐Aldrich, St. Louis, MO, USA), LATS1/2 (1:500, cat. ab70565), pLATS1/2 (phospho T1079 + T1041; 1:500, cat. ab111344; Abcam), E‐cadherin (1:500, cat. 610181) and N‐cadherin (1:1,000, cat. 610920; BD Transduction Laboratories, Lexington, KY, USA). After incubation with anti‐mouse (1:2,000, E030110‐01) or anti‐rabbit (1:2,000, E030120‐01) IgG antibodies (EarthOx LLC, San Francisco, CA, USA) at 37°C for 2 hrs, the protein bands were visualized using enhanced chemiluminescence (ECL; Thermo Fisher Scientific, Waltham, MA, USA) and quantified using a BioImaging Systems platform (UVP, Upland, CA, USA). Relative protein levels were calculated using GAPDH as the loading control.

### Real‐time quantitative RT‐PCR

Total RNA was extracted from cells using TRIzol reagent according to the manufacturer's protocol. Next, cDNA was synthesized from 1 μg of total RNA by a PrimerScript RT Reagent kit (TaKaRa, Dalian, China). Real‐time quantitative RT‐PCR was performed using a SYBR green PCR master mix in an Applied Biosystems StepOne and StepOne‐Plus Real‐Time PCR system (Applied Biosystems, Foster City, CA, USA). The primers used for real‐time PCR were shown in Table S1. The gene expression ΔCt values of mRNA from each sample were calculated by normalizing with the reference gene 18s. All experiments were repeated in triplicate to confirm the findings.

### Matrigel cell invasion assay

Matrigel cell invasion assays were performed using 8‐μm polycarbonate membranes (Corning, Acton, MA, USA) according to the manufacturer's instructions. Cell suspension (5 × 10^5^ cells; 100 μl) was added to the upper chamber, while the lower chamber was filled with RPMI‐1640 medium containing 10% foetal calf serum. The plates were incubated for 24 hrs, and then cells that had invaded to the lower membrane surface were fixed in methanol for 30 min. and stained with haematoxylin (Sigma). For each filter, the numbers of cells that invaded to the lower surface of the membrane were counted in five randomly selected fields of view at 200 × magnification using a Nikon E200 light microscope (Nikon, Melville, NY, USA). Mean cell numbers were calculated from the data obtained from each experiment repeated three times.

### Statistical analysis

SPSS statistical software (standard version 16.0; SPSS, Chicago, IL, USA) was used to perform the Pearson chi‐squared test, Kaplan–Meier analysis and log‐rank tests. GraphPad Prism 5 software (GraphPad Software, Inc., San Diego, CA, USA) was used to perform two‐tailed Student's *t*‐tests. *P* < 0.05 was considered statistically significant.

## Results

### Down‐regulation of WWC2 protein expression is clinically significant in HCC

**Figure 1:**
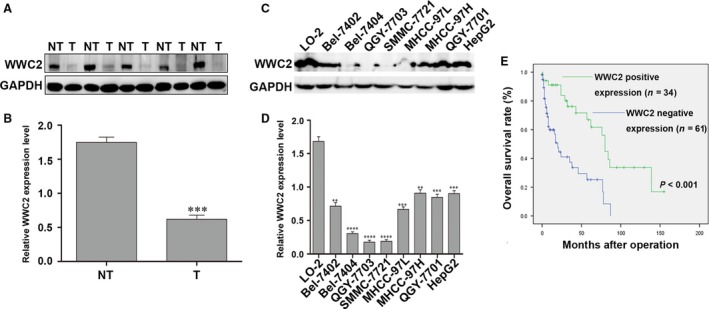
WWC2 is down‐regulated and clinically significant in hepatocellular carcinoma (HCC). (**A, B**) Representative Western blot (**A**) and quantitative analysis (**B**) of WWC2 expression in 24 paired HCC (T) and matched adjacent non‐tumour tissues (NT); values are mean ± standard error of the mean. WWC2 protein expression was significantly lower in HCC tissues than in normal tumour‐adjacent tissues (***P < 0.001, *t*‐test). (**C, D**) Western blot (**C**) and quantitative analyses (**D**) of WWC2 expression in LO‐2 cells and eight HCC cell lines (***P < 0.001, *t*‐test). (**E**) Kaplan–Meier overall survival curves for 95 patients with HCC stratified by WWC2 expression; positive WWC2 expression was associated with better overall survival (***P < 0.001, log‐rank test).

Initially, we performed immunohistochemistry on 95 paired tumour samples and paired normal tumour‐adjacent samples obtained during liver resection from patients with HCC. WWC2 expression was detected in 93.7% (89/95) of the matched non‐cancerous tissue samples, whereas only 35.8% (34/95) of the HCC specimens demonstrated positive WWC2 expression (*P* < 0.001). Furthermore, Western blotting of 24 paired HCC tissues and adjacent non‐tumour tissues confirmed WWC2 protein expression was significantly decreased in HCC tissues compared with the paired adjacent non‐tumour tissues (*P* < 0.001; Fig. 1A and B). Retrospective clinical association analysis using the Pearson chi‐squared test revealed decreased WWC2 expression in HCC was positively associated with tumour size (*P* = 0.030), venous infiltration (*P* = 0.006) and advanced tumour stage (TNM stage III + IV; *P* = 0.001; Table 1).

To further confirm WWC2 is down‐regulated in HCC, we performed Western blotting of eight HCC cell lines and the normal liver cell line LO2. The expression of WWC2 was significantly lower in all eight HCC cell lines than LO2 cells (*P* < 0.001; Fig. 1C and D).

### Positive WWC2 expression correlates with better 5‐year survival in HCC

Next, we examined the prognostic value of WWC2 for overall survival in HCC. Kaplan–Meier survival curves (Fig. 1E) revealed positive WWC2 expression was associated with significantly better overall survival (log‐rank = 15.836; *P* = 0.000); overall survival was 45.0% for WWC2‐negative HCC compared to 52.9% for WWC2‐positive HCC. Multivariate Cox regression indicated that, in addition to tumour stage (TNM stage III + IV; *P* = 0.015) and the number of tumour nodules (*P* = 0.041), WWC2 expression was an independent prognostic factor for overall survival in HCC (*P* = 0.003; Table 2).

**Table 2:** Multivariate Cox regression analysis of factors associated with overall survival in hepatocellular carcinoma

Variable	OS		*P‐*value
	HR	95% CI	
Age	0.584	0.301–1.136	0.113
Gender	1.260	0.449–3.534	0.660
HBV	0.917	0.286–2.940	0.884
Cirrhosis	1.524	0.549–4.232	0.418
Tumour size	0.709	0.324–1.551	0.389
Serum AFP	0.564	0.277–1.148	0.114
No. of tumour nodules	2.459	1.037–5.829	0.041^a^
Differentiation	1.612	0.914–2.842	0.099
Capsule invasion	0.690	0.339–1.407	0.307
Venous infiltration	0.740	0.336–1.626	0.453
TNM stage	2.686	1.216–5.934	0.015^a^
WWC2	0.311	0.143–0.679	0.003^a^

OS, overall survival; HBV, hepatitis B virus; AFP, alpha‐fetoprotein; TNM, tumour‐node‐metastasis; HR, hazard ratio; CI, confidence interval.

a Statistically significant.

### Overexpression of WWC2 inhibits the invasion of HCC cells

**Figure 2:**
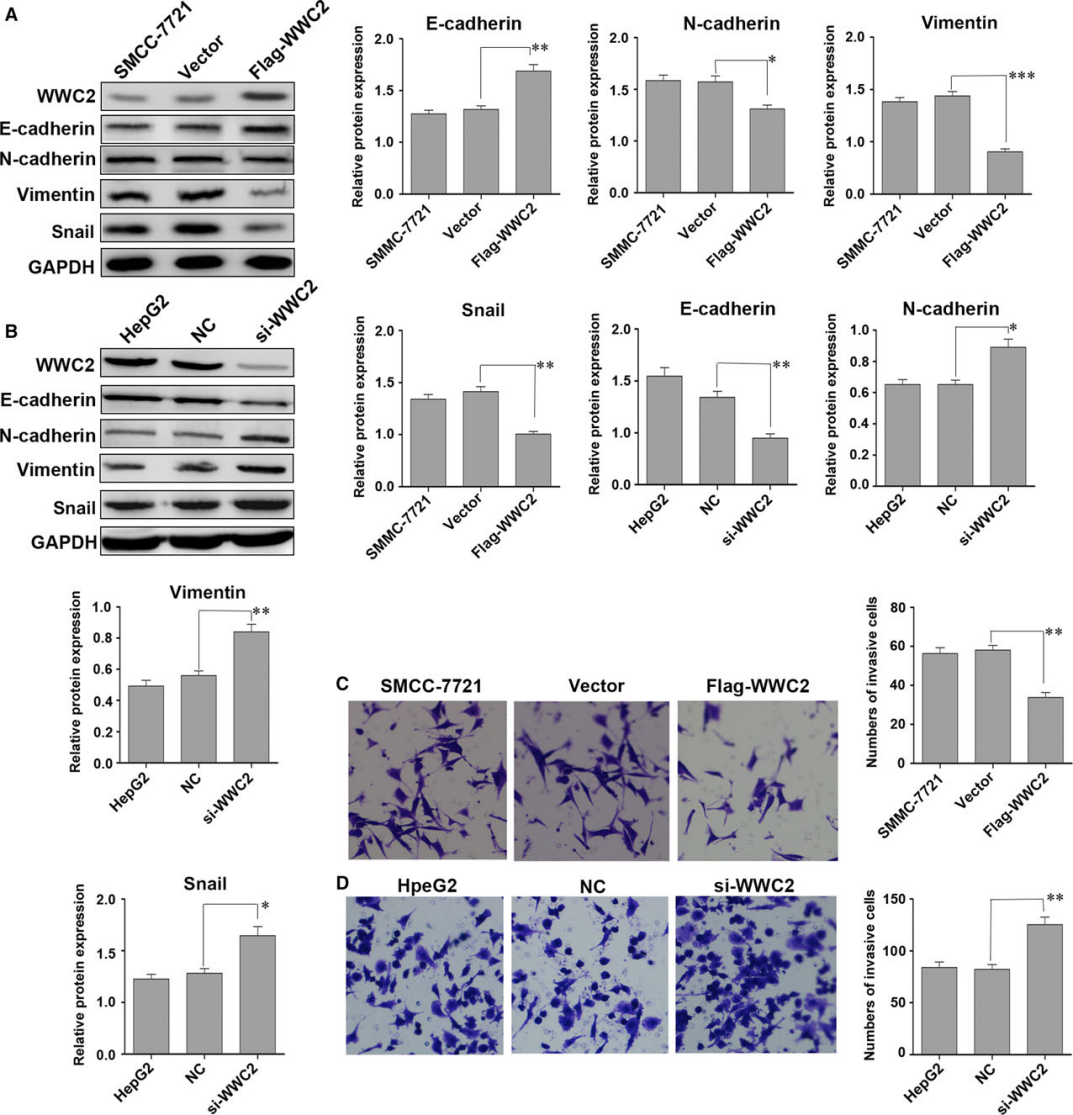
Overexpression of WWC2 reduces the invasive ability of hepatocellular carcinoma (HCC) cells. (**A**) SMCC‐7721 cells transiently transfected with the WWC2 plasmid showed increased E‐cadherin expression and decreased N‐cadherin, vimentin and snail expression. (**B**) Knockdown of *WWC2* decreased E‐cadherin expression and increased N‐cadherin, snail and vimentin expression in HepG2 cells. (**C**) Transient transfection of WWC2 into SMCC‐7721 cells decreased cell invasive ability (**P < 0.01). (**D**) Knockdown of *WWC2* enhanced the invasive ability of HepG2 cells (**P < 0.01, *t*‐test).

To investigate the effect of WWC2 on invasion in HCC, we selected cells expressing low levels (SMMC‐7721 cells) and high levels of WWC2 (HepG2 cells) for further studies. Western blot analysis demonstrated overexpression of WWC2 using an expression vector increased E‐cadherin expression and decreased N‐cadherin, vimentin and snail expression in SMMC‐7721 cells (*P* = 0.006, *P* = 0.02, *P* = 0.0005, *P* = 0.0015, respectively; Fig. 2A). Conversely, knockdown of *WWC2* using a siRNA decreased E‐cadherin expression and increased N‐cadherin, vimentin and snail expression in HepG2 cells (*P* = 0.005, *P* = 0.016, *P* = 0.007, *P* = 0.021, respectively; Fig. 2B).

Moreover, overexpression of WWC2 reduced the invasive ability of SMMC‐7721 cells (52.0 ± 1.20 *versus* 33.33 ± 1.50, *P* < 0.001; Fig. 2C), whereas knockdown of *WWC2* enhanced the invasive ability of HepG2 cells (18.0 ± 0.82 *versus* 39.6 ± 0.65, *P* < 0.01; Fig. 2D).

### Positive WWC2 expression correlates negatively with nuclear YAP protein in HCC tissues

**Figure 3:**
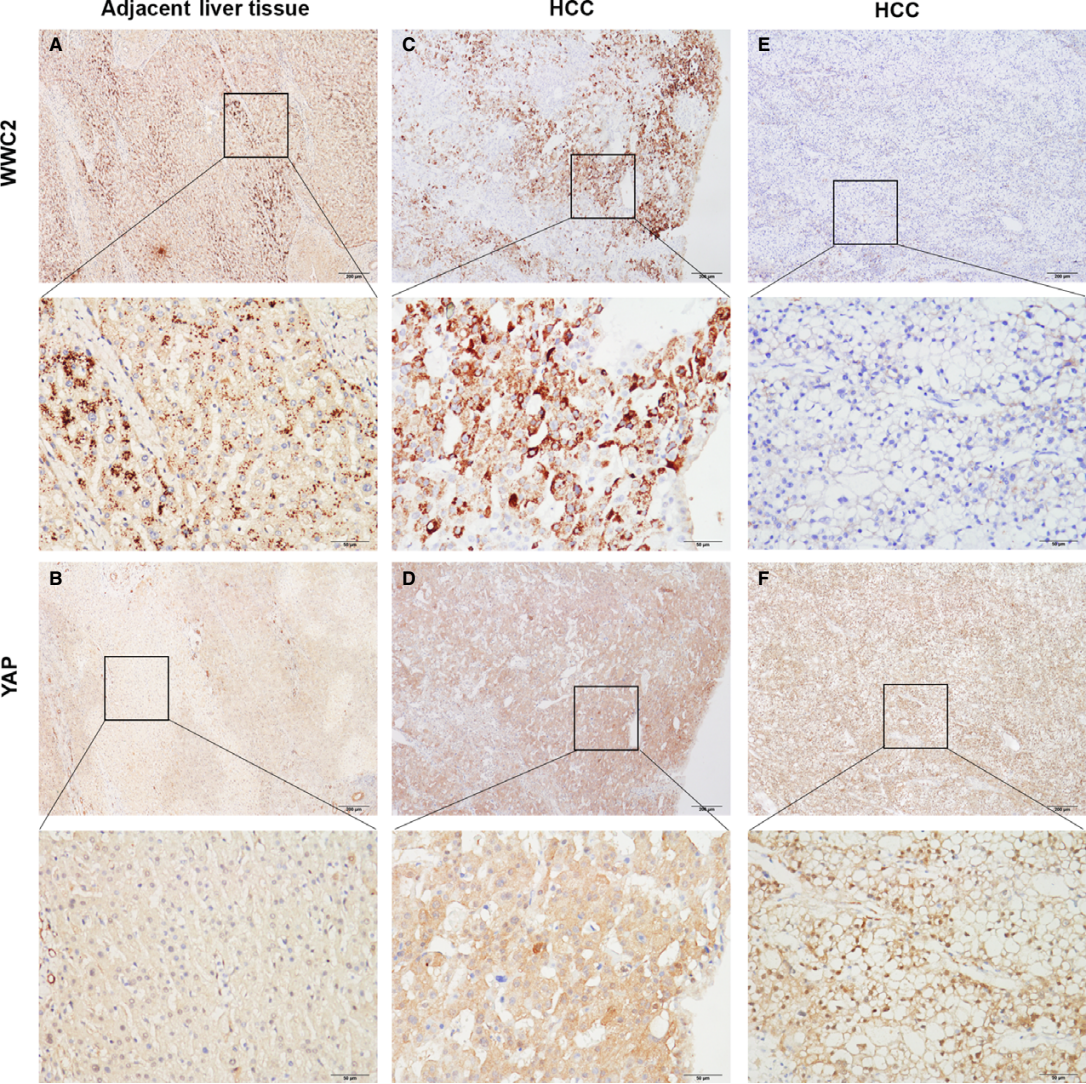
WWC2 is down‐regulated and correlates inversely with Yes‐associated protein (YAP) nuclear expression in human hepatocellular carcinoma (HCC). (**A, B**) Representative photomicrographs of WWC2 immunohistochemistry in HCC tissues and the matched adjacent non‐tumour tissues. In the adjacent liver tissues, WWC2 expression was generally positive (**A**) with cytoplasmic YAP expression (**B**). (**C, D**) In cases of HCC with high WWC2 protein expression (**C**), low nuclear YAP protein expression and strong cytoplasmic YAP protein expression were observed (**D**). (**E, F**) In cases of HCC with low WWC2 protein expression (**E**), low cytoplasmic YAP protein expression and strong nuclear YAP protein expression were observed (**F**).

As the transcriptional co‐activator YAP can be located in both the cytoplasm and nucleus 12, 27, we examined the associations between WWC2 expression and cytoplasmic and nuclear YAP expression in HCC. Nuclear YAP expression was significantly higher in HCC than the paired non‐cancerous tissues [72.6% (69/95) *versus* 32.6% (31/95); *P* = 0.027]. On the contrary, cytoplasmic YAP expression was significantly lower in HCC than non‐cancerous tissues [50.5% (48/95) *versus* 67.4% (64/95); *P* = 0.006]. Immunohistochemistry scores were generated for quantitative analysis of WWC2 and YAP expression in the 95 HCC samples. WWC2 expression correlated negatively with cytoplasmic YAP expression (*P* = 0.014) and positively with nuclear YAP expression (*P* = 0.045; Fig. 3C‐F and Table 3).

**Table 3:** Associations between WWC2 expression and nuclear and cytoplasmic Yes‐associated protein (YAP) expression in hepatocellular carcinoma

	Patients	WWC2 expression		X^2^	*P‐*value
		Negative	Positive		
Nuclear YAP expression					
Negative	26	11	15	7.473	0.006^a^
Positive	69	50	19		
Cytoplasmic YAP expression					
Negative	47	36	11	6.209	0.013^a^
Positive	48	25	23		

HCC, hepatocellular carcinoma.

a Statistically significant.

### WWC2 negatively regulates Hippo signalling in HCC by activating LATS2, which in turn phosphorylates YAP

**Figure 4:**
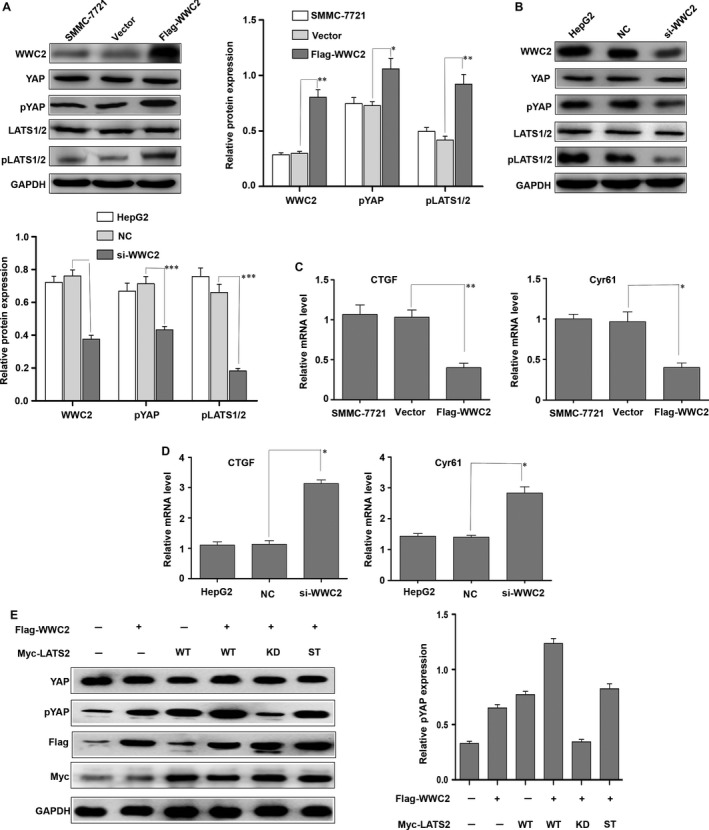
Overexpression of WWC2 negatively regulates the Hippo pathway in hepatocellular carcinoma (HCC) cells. Western blot analysis of Yes‐associated protein (YAP) protein levels in HCC cells transfected with WWC2 or control vector. (**A**) Quantitative Western blot analysis showing transient transfection of SMCC‐7721 cells with the WWC2 plasmid increased the levels of p‐LATS1/2 (**P < 0.01) and p‐YAP (*P < 0.05). (**B**) Quantitative Western blot analysis showing transient transfection of HepG2 cells with the siRNA targeting *WWC2* decreased the levels of p‐LATS1/2 (**P < 0.001) and p‐YAP (*P < 0.001). (**C**)Real‐time quantitative RT‐PCR analysis showing transient transfection of SMCC‐7721 cells with the WWC2 plasmid decreased the mRNA levels of connective tissue growth factor (CTGF) (**P < 0.01) and Cyr61(*P < 0.05). (**D**) Real‐time quantitative RT‐PCR analysis showing transient transfection of HepG2 cells with the siRNA targeting *WWC2* increased the mRNA levels of CTGF (*P < 0.05) and Cyr61(*P < 0.05). (**E**) SMCC‐7721 cells were transfected WWC2 with WT LATS2 or various LATS2 mutants. Cell lysates were prepared at 48 hrs post‐transfection and probed with the indicated antibodies. WT, wild type; KD, kinase‐dead form (K697R); ST, LATS2 with both S872A and T1041A mutations.*P < 0.05, **P < 0.01, ***P < 0.001, ****P < 0.0001.

To further characterize the function of WWC2 and to explore the relationship between WWC2 and the Hippo signalling pathway in HCC, we analysed the phosphorylation status of LATS1/2 and YAP, major components of the Hippo pathway. Western blot analysis revealed phosphorylation of both LATS1/2 and YAP significantly increased at 48 hrs in HCC cells transfected with the WWC2 overexpression plasmid (*P* = 0.007 and *P* = 0.0045, respectively, compared to cells transfected with vector control plasmid and untreated cells; Fig. 4A). Conversely, the levels of p‐LATS1/2 and p‐YAP were lower in HepG2 cells transfected with the *WWC2* siRNA than cells transfected with the NC‐siRNA (*P* = 0.005 and *P* = 0.031; Fig. 4B). In addition, it has been reported that several genes are regulated by YAP, including connective tissue growth factor (CTGF) and Cyr61^28^, ^29^, ^30^, ^31^. We next examined the mRNA levels of the two genes by real‐time quantitative RT‐PCR. As shown in Figure 4C, overexpressing WWC2 was sufficient to suppress the expression of CTGF and Cyr61 at the mRNA level. On the contrast, HepG2 cells transfected with si‐WWC2 showed an increased mRNA level of CTGF and Cyr61(Fig. 4D). Together, these results suggest WWC2 regulates the Hippo pathway in HCC by phosphorylating LATS1/2 and YAP.

LATS1/2 can inactivate YAP by phosphorylating the serine 127 residue of YAP 32, 33. To confirm WWC2 promotes phosphorylation of YAP by activating the LATS1/2 kinases in HCC, we cotransfected SMMC‐7721 cells with the WWC2 expression plasmid and WT LATS2 or various LATS2 mutants. As shown in Figure 4C, cotransfection of the kinase‐dead form of LATS2 (K697R mutant) markedly blocked WWC2‐stimulated YAP phosphorylation (compare lane 5 with lanes 2 to 4; *P* = 0.001, *P* = 0.0003, *P* = 0.0001, respectively). The non‐phosphorylatable mutant form (LATS2 with both S872A and T1041A mutations) partially blocked YAP phosphorylation compared to the LATS2‐KD mutant (*P* = 0.0006; Fig. 4C). Thus, our data indicate WWC2 negatively regulates the Hippo pathway in HCC by activating LATS2, which in turn phosphorylates the transcriptional co‐activator YAP and prevents its nuclear translocation.

### WWC2 suppresses the invasive ability of HCC cells by negatively regulating Hippo signalling

**Figure 5:**
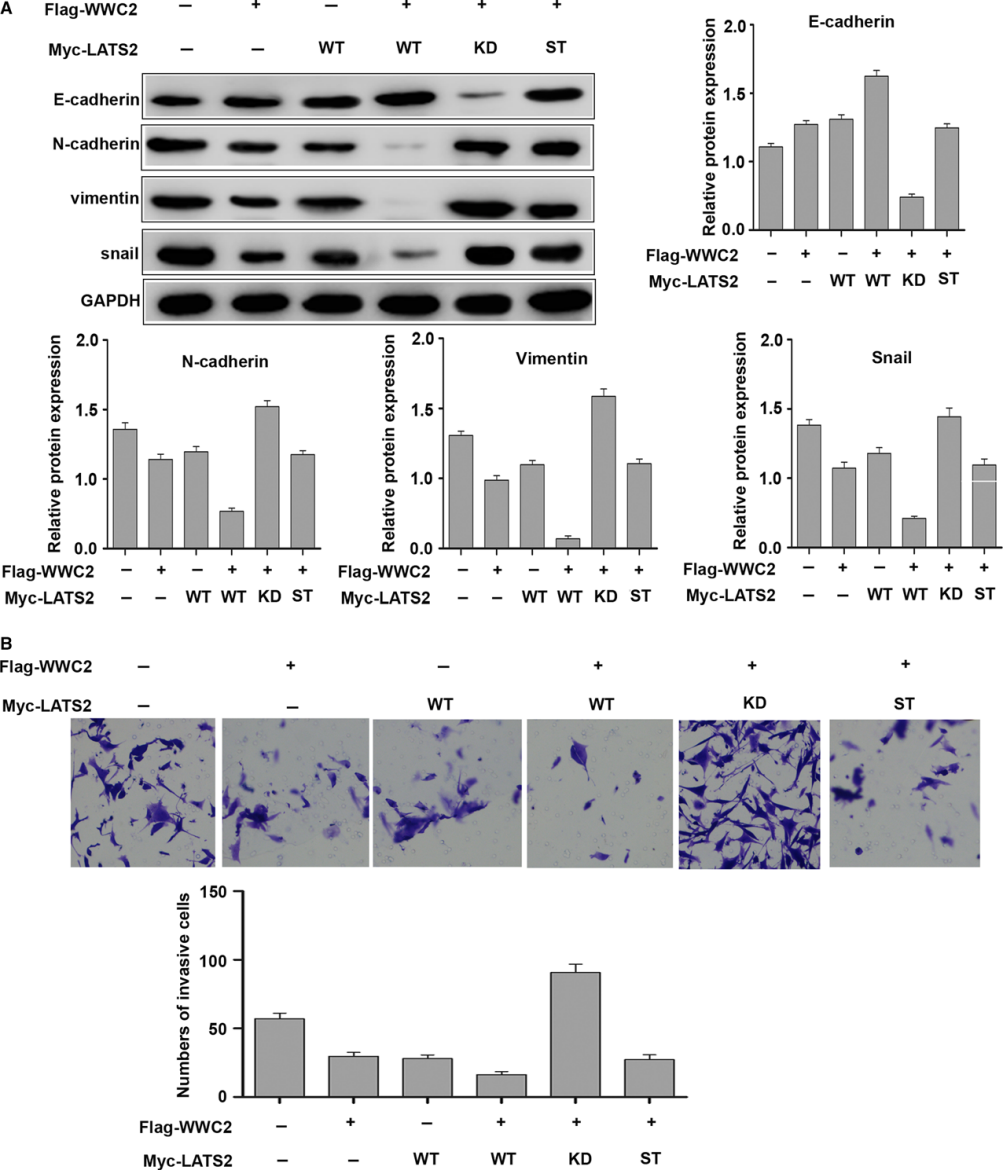
WWC2 suppresses the invasive ability of hepatocellular carcinoma (HCC) cells by activating LATS2, which in turn phosphorylates Yes‐associated protein (YAP). (**A**) Western blot analysis showing cotransfection with WWC2 and LATS2‐KD decreased E‐cadherin expression and increased N‐cadherin, vimentin and snail expression in SMMC‐7721 cells compared to cotransfection with LATS2‐WT (*P* = 0.0001, *P* = 0.0002, *P* = 0.0001, *P* = 0.0004, respectively). The LATS2‐ST partially inhibited the epithelial mesenchymal transition (EMT) compared to the LATS2‐KD (*P* = 0.0002 for E‐cadherin, *P* = 0.0028 for N‐cadherin, *P* = 0.0014 for vimentin and *P* = 0.0111 for snail, respectively). (**B**) Cotransfection of the LATS2‐KD also reduced cell invasion compared to SMMC‐7721 cells cotransfected with LATS2‐WT (*P* = 0.0023). The LATS2‐ST partially inhibited cell invasion, as compared with the LATS2‐KD mutant (*P* = 0.0012). WT, wild type; KD, kinase‐dead form (K697R); ST, LATS2 with both S872A and T1041A mutations.

To determine whether the ability of WWC2 to regulate Hippo signalling affects invasion in HCC, SMMC‐7721 cells, which express low levels of WWC2, were cotransfected flag‐WWC2 and WT LATS2 or various LATS2 mutants. Western blot analysis demonstrated cotransfection of the kinase‐dead form LATS2 mutant (LATS2‐KD) decreased E‐cadherin expression and increased N‐cadherin, vimentin and snail expression, compared to SMMC‐7721 cells cotransfected with LATS2‐WT (*P* = 0.0001, *P* = 0.0002, *P* = 0.0001, *P* = 0.0004, respectively; Fig. 5A). Transfection of the non‐phosphorylatable mutant form of LATS2 (LATS2 with both S872A and T1041A mutations) partially inhibited the EMT compared with the LATS2‐KD mutant (*P* = 0.0002 for E‐cadherin, *P* = 0.0028 for N‐cadherin, *P* = 0.0014 for vimentin and *P* = 0.0111 for snail, respectively; Fig. 5A). Cotransfection of LATS2‐KD also reduced the invasive ability of SMMC‐7721 cells compared to cotransfection of LATS2‐WT (*P* = 0.0023; Fig. 5B). Moreover, the non‐phosphorylatable mutant form (LATS2 with both S872A and T1041A mutations) partially inhibited invasion compared to cells transfected with the kinase‐inactive LATS2‐KD mutant (*P* = 0.0012; Fig. 5B). Taken together, these data indicate that WWC2 suppresses the EMT and invasion in HCC cells by negatively regulating the Hippo signalling pathway.

## Discussion

WWC family proteins negatively regulate Hippo signalling by activating the LATS1/2 kinases, which in turn phosphorylate the transcriptional co‐activator YAP and prevents its nuclear translocation in mammalian cells 21, 25, 34, 35. Wennmann *et al*. subsequently verified endogenous LATS1/2 kinases bind to all types of WWC proteins in HEK293 cells 22. In drosophila, loss of KIBRA function increased proliferation, reduced apoptosis and regulated Hippo signalling targets; these changes are the typical phenotypes associated with loss of function of Hippo components 36, 37, 38. However, these studies focused on Kibra/WWC1, and the biological effects and molecular mechanisms of action of WWC2 in cancer remained unclear. This study indicates WWC2 negatively regulates the Hippo pathway in HCC, implying WWC2 may represent a candidate target protein for cancer therapeutics.

First, we demonstrated WWC2 protein expression was significantly down‐regulated in HCC compared with the matched adjacent non‐tumour tissues, and the absence of WWC2 expression was significantly associated with larger tumour size, venous infiltration and advanced TNM tumour stage. Moreover, positive WWC2 expression was associated with significantly better 5‐year overall survival and WWC2 was an independent prognostic factor for 5‐year overall survival, which suggests WWC2 functions as a tumour suppressor in HCC. These findings indicate assessment of WWC2 expression may help to determine the prognosis of patients with HCC. Furthermore, we confirmed that WWC2 suppresses tumour progression by inhibiting the invasive ability of HCC cells.

WWC2 has been reported to be involved in Hippo signalling 22. As YAP is a pivotal effector of the Hippo pathway, we examined the correlation between WWC2 and YAP protein expression in HCC tissues. The function of the transcriptional co‐activator YAP varies depending on its subcellular localization 12, 27. WWC2 expression correlated negatively with nuclear YAP expression and positively with cytoplasmic YAP expression in HCC. This data further indicate WWC2 functions as tumour suppressor by acting as an upstream regulator of YAP in the Hippo signalling pathway, and this relationship plays a role in the development of HCC; further studies are required to clarify the role of WWC2 in the development of cancer.

WWC2 can suppress cell proliferation and organ growth by activating members of the Hippo tumour suppressor pathway, including LATS1/2; Wennmann *et al*. have verified WWC2 directly binds to LATS1/2 *via* their WW domains, and phosphorylates and activates LATS1/2 22. Activated LATS1/2 phosphorylate YAP, which inhibited cell proliferation in mammalian cells 38, 39, 40. In confirmation that WWC2 activates the Hippo pathway in HCC, overexpression of WWC2 in SMMC‐7721 cells (which express low levels of WWC2) increased phosphorylation of both LATS and YAP, which has been previously shown to negatively regulate the Hippo pathway 11, 41, 42. The latter study also revealed HCC cells transfected with the WWC2 overexpression plasmid decreased the mRNA level of Cyr61 and CTGF, both which are the target genes of Hippo pathway. All these very well‐confirmed overexpressing WWC2 negatively regulate the Hippo pathway.

In agreement with previous studies 22, we confirmed WWC2 phosphorylates LATS1/2, which in turn phosphorylates YAP in HCC cells. Indeed, cotransfection of WWC2 with a kinase‐inactive form (LATS2‐KD, K697R mutant) or non‐phosphorylatable mutant form (LATS2 with both S872A and T1041A mutations) provided a relatively objective model to verify WWC2 regulates YAP phosphorylation by activating the LATS2 kinase. Phosphorylated YAP cannot translocate to the nucleus, where it serves as a co‐activator for the TEA‐domain family member (TEAD) group of DNA‐binding transcription factors 22. Moreover, overexpression of WWC2 in SMMC‐7721 cells decreased nuclear YAP expression, consistent with our immunohistochemistry results that WWC2 expression correlated negatively with nuclear YAP expression in HCC.

In conclusion, we demonstrate that down‐regulation of WWC2 is associated with the clinicopathological features of advanced HCC. Negative WWC2 expression was an independent prognostic factor for poor survival in HCC. Moreover, WWC2 suppresses the invasion and metastatic potential of HCC cells by negatively regulating the Hippo signalling pathway by phosphorylating LATS2, which in turn phosphorylates and inhibits nuclear translocation of the transcriptional co‐activator YAP. These findings indicate WWC2 may represent a potential therapeutic target for HCC.

## Conflict of Interest

The authors declare that they have no conflict of interests.

## Supporting information


**Table S1** Primer sequences for quantitative real‐time RT‐PCR.
