# Supplementary material for: WWC2 is an independent prognostic factor and prevents invasion via Hippo signalling in hepatocellular carcinoma
Source: J Cell Mol Med. 2017 Aug 16;21(12):3718–29. doi: 10.1111/jcmm.13281 (PMC5706493; doi:10.1111/jcmm.13281)
Supplement: Supplementary file 1 — Table S1 Primer sequences for quantitative real‐time RT‐PCR. [file JCMM-21-3718-s001.docx]

**Supplementary table 1 Primer sequences for quantitative real-time RT-PCR**

| Gene product | Primer Sequence |  |
| --- | --- | --- |
| CTGF | Forward  Reverse | CAGCATGGACGTTCGTCTG  AACCACGGTTTGGTCCTTGG |
| Cyr61 | Forward  Reverse | CTCGCCTTAGTCGTCACCC  CGCCGAAGTTGCATTCCAG |
| 18s | Forward  Reverse | AATAGCCTTTGCCATCAC  CGTTCCACCTCATCCTC |
